# Pattern analysis of vegan eating reveals healthy and unhealthy patterns within the vegan diet

**DOI:** 10.1017/S136898002100197X

**Published:** 2022-05

**Authors:** Catherine T Gallagher, Paul Hanley, Katie E Lane

**Affiliations:** Research Institute for Sport and Exercise Sciences, I. M. Marsh Campus, Liverpool John Moores University, Barkhill Road, Aigburth, Liverpool L17 6BD, UK

**Keywords:** Dietary pattern analysis, Vegan diet, Convenience, Traditional, Healthy, Ultraprocessed foods

## Abstract

**Objective:**

This study aimed to identify the types of foods that constitute a vegan diet and establish patterns within the diet. Dietary pattern analysis, a key instrument for exploring the correlation between health and disease, was used to identify patterns within the vegan diet.

**Design:**

A modified version of the EPIC-Norfolk FFQ was created and validated to include vegan foods and launched on social media.

**Setting:**

UK participants, recruited online.

**Participants:**

A convenience sample of 129 vegans voluntarily completed the FFQ. Collected data were converted to reflect weekly consumption to enable factor and cluster analyses.

**Results:**

Factor analysis identified four distinct dietary patterns including: (1) convenience (22 %); (2) health conscious (12 %); (3) unhealthy (9 %) and (4) traditional vegan (7 %). Whilst two healthy patterns were defined, the convenience pattern was the most identifiable pattern with a prominence of vegan convenience meals and snacks, vegan sweets and desserts, sauces, condiments and fats. Cluster analysis identified three clusters, cluster 1 ‘convenience’ (26·8 %), cluster 2 ‘traditional’ (22 %) and cluster 3 ‘health conscious’ (51·2 %). Clusters 1 and 2 consisted of an array of ultraprocessed vegan food items. Together, both clusters represent almost half of the participants and yielding similar results to the predominant dietary pattern, strengthens the factor analysis.

**Conclusions:**

These novel results highlight the need for further dietary pattern studies with full nutrition and blood metabolite analysis in larger samples of vegans to enhance and ratify these results.

Over half a million people in the UK (≈1 % of the population) follow a vegan diet where all animal sources are substituted with plant-based alternatives. Veganism quadrupled between 2014 and 2019 in the UK^(1)^ with 600 000 vegans reported in 2019^(2,3)^, while the popularity in vegan diets continues to grow worldwide^(4)^. The food industry is responding to this by producing more processed vegan food and drink products than ever before^(2,5)^. In January 2021, ‘Veganuary’ saw over 440 000 people in the UK committing to a vegan diet^(6)^, raising the profile of plant-based eating which has been associated with a range of health benefits^(7)^.

It is reported that a well-planned vegan diet can meet all the nutritional requirements necessary for health^(8)^. There is still some debate, however, about the nutritional quality of vegan diets and the risk of nutritional deficiencies, notably some key micronutrients such as vitamin B_12_, vitamin D, Fe, Ca, iodine, *n*-3, Se and Zn in poorly adapted or non-fortified vegan diets^(9)^. In dietary terms, a traditional vegan diet refers to a diet that omits all products derived wholly or partly from animal origin. The diet focuses more on wholegrains, pulses, fruit and vegetables^(10)^. It remains unclear if modern vegan dietary adaptation methods can deliver the same health advantages as traditional vegan diets. For example, if vegans are choosing ultraprocessed vegan products over more natural plant-based alternative sources, could this compromise the overall quality of the vegan diet?^(11)^. By way of definition, ultraprocessed foods refer to products mostly or entirely formulated from substances derived from foods that typically contain little or no whole foods^(12)^. These products are usually high in saturated fat, sugar and salt. The majority of these food items are also considered poor sources of protein, fibre and micronutrients^(13,14)^. Studies over the past two decades have provided important information on the diet quality of various types of vegetarians, but no single study has addressed the quality of specific vegan diets. Orlich *et al.* ^(7)^ reveal Adventist vegans consumed the lowest amounts of foods and snacks high in added sugars and saturated fats, in comparison with non-vegetarians and other vegetarian groups. This argument is consistent with much of the literature surrounding vegan diets^(15−18)^. However, the main weakness with this research is that it is outdated and perhaps not considering the increasing variety of processed food and drinks that are now available to vegans. In 2018, the UK developed more vegan products than any other nation^(5)^. Popular UK supermarkets are reacting by producing vegan wines with a pledge to ensure their full range is suitable for vegans in the coming years^(19)^. In 2019, Galaxy launched a vegan Mars bar in the UK^(20)^, and in 2020, Mc Donald’s launched its first vegan meal^(21)^. Thus, the production of vegan alternatives including vegan snacks and fast foods is prevalent and represents one of the main product development trends within the food and retail industry. However, many of these food items can be high in saturated fats and sugars and if eaten regularly may pose a risk to health. Therefore, a review of current vegan dietary patterns is urgently required to address these uncertainties.

Several studies have evaluated the dietary patterns of omnivores, pesco, lacto, ovo and semi-vegetarians in comparison with vegan diets^(22−26)^, but none to date has subjected vegan diets to dietary pattern analysis. It is important to establish whether the increased availability of processed vegan replacements for animal-based products is leading to habitual consumption of an array of ultraprocessed foods. The methodology for this unique study includes an innovative dietary pattern analysis of vegan diets. Dietary pattern analysis offers an effective way of understanding the diverse eating patterns within vegan diets by evaluating methods of adaptation and substitution^(27)^. It was hypothesised that some vegan diets would incorporate a range of food groups representing a traditional well-planned vegan diet. This was expected to be the most common dietary pattern. The vegan food industry has evolved; therefore, it was predicted that a convenience style eating pattern could also emerge, representing a small proportion of the participants.

This study aimed to identify patterns within the vegan diet by establishing the everyday foods that vegans are choosing to consume enabling an evidence-based evaluation of the vegan diet.

## Methods

### FFQ

A FFQ was created using LJMU-approved Online survey tool, an online food questionnaire creator, to enable the provision of a validated interactive dietary assessment tool^(28)^. The validated EPIC-Norfolk FFQ^(29)^ was modified to include questions representative of foods and drinks suitable for vegans. Adaptation followed methods used by Dyett *et al.* ^(30)^ in their evaluation of a validated FFQ for self-defined vegans in the USA. Vegan food items available in the UK were identified from mainstream UK supermarkets and vegan UK forums. A collection of naturally vegan food products and newly emerging ultraprocessed vegan products were included in the FFQ. Ten vegan volunteers in a UK university who met the study criteria took part in an initial pilot study. Feedback from the volunteers was taken on board to further modify the vegan FFQ. To further enhance validation of the vegan-adapted FFQ, a focus group of Health and Care Professions Council registered dietitians in the UK were then consulted. Modifications and additions to the food groups were made accordingly based on the dietitians comments to generate the finalised version of the vegan-adapted FFQ (see online supplemental material S1). Questionnaire instructions stated that the FFQ must reflect dietary habits over the past month, and therefore, participants must have been following a vegan diet for at least 1 month. Further questions were included such as motivations for adopting vegan lifestyle, age, length of time vegan, cooking skills and supplement use to ensure evaluation of factors influencing diet choice and nutritional knowledge^(31)^.

### Recruitment

Online social media accounts (Instagram and Facebook) were used to recruit subjects. The FFQ was launched on social media accounts in the UK. The recruitment team asked for vegans in the UK to complete and share the FFQ. In order to reduce bias, participants’ involvement in this study was voluntary. Participants gave informed consent prior to completing the voluntary FFQ. Inclusion criteria required participants to be living in the UK and aged over 18 years, so only adults could take part. Participants were also required to have followed a vegan diet for at least 1 month. This allowed specific dietary patterns to be captured.

### Statistical analysis

Statistical analyses were performed using IBM SPSS (version 26.0; SPSS Inc.) and Microsoft Excel 2013. Data screening and cleaning was conducted to check for any outliers and errors on the categorical and continuous variables. Descriptive statistics such as frequencies and percentages were calculated for characterisation of the participants (i.e. gender, age groups and length of time vegan). Statistical tests were used to calculate the significance of error.

### Data screening

Selected frequency of consumption for each food in the FFQ was coded to reflect how often each item was consumed per week for dietary pattern analysis as followed: NEVER or less than once/month 0, 1–3/month, once a week, 2–4/week, 5–6/week, once a day, 2–3/d, 4–5/d, 6+/d. This design was taken from the validated EPIC-Norfolk FFQ, which has also been used in other studies^(32,33)^. Two methods were used to classify the individual food items before applying factor and cluster analyses. In the first instance, the food and drink items were combined and collapsed into thirty food groups and in the second twenty food groups (Table 1), respectively, with similar nutrient profiles, similarly to previous research by Ashby-Mitchell *et al.* ^(34)^.

**Table 1:** Food groups and food items included in the analysis of the FFQ cohort

Food groups 1	Food groups 2 (variables)	Definition and content
1. Legumes & nuts 2. Meat alternatives	1. Protein alternatives to meat & fish	Soya, Tempeh, Tofu, silken tofu, lentils, pulses, nuts, falafel
3. Meat-free processed alternatives 4. Fish alternatives	2. Processed meat alternatives	Vegan nuggets, burgers, bacon, sausage, no fish fingers, ham slices, turkey slices, chicken slices, meat-free mince, vegan chorizo
5. Vegan sandwiches 6. Vegan wraps 7. Ready-prepared foods	3. Convenience meals & snacks	Garlic bread, pizza, sausage rolls, chips, ready prepared mash, selection of pre-made vegan sandwiches & wraps, ready meals, Not-zarrella sticks, French fries
8. Fresh fruit 9. Tinned fruit 10. Dried fruit	4. Fruit	Apples, pears, oranges, grapefruit, bananas, grapes, melon, peaches, strawberries, avocado, tinned fruit, dried fruit
11. Vegetables 12. Soup	5. Vegetables	Carrots, spinach, broccoli, Brussel sprouts, cabbage, peas, green beans, courgettes, cauliflower, parsnips, leeks, onions, garlic, mushrooms, sweet peppers, beansprouts, green salad, mixed vegetables, watercress, tomatoes, sweetcorn, beetroot, coleslaw, vegetable soup, rainbow rice
13. Starchy carbohydrates	6. Refined grains	White bread, scones, crackers, pitta, sugary cereal, plain cereal, white rice, pasta, tinned pasta, noodles, lasagne, cereals (except high fibre options)
14. High-fibre carbohydrates	7. Wholegrains	Brown bread, wholemeal bread, porridge, all bran, wholegrain cereals, brown rice, wholemeal pasta, wild rice
15. White potatoes 16. Sweet potatoes	8. Potatoes	Boiled potatoes, roast potatoes, sweet potatoes, home-made mash, baked potatoes, baby potatoes
17. Plant-based milks 18. Vegan cheese 19. Vegan yoghurts	9. Dairy alternatives	Oat milk, soya milk, almond milk, rice milk, hazelnut milk, coconut milk hemp, pea milk, Nutritional yeast, vegan hard cheese, Yoghurt alternatives,
20. Fats and oils	10. Fats and oils	Vegan butter spreads, pesto, peanut butter, olive oil, sunflower oil, coconut oil, avocado oil, canola oil, sunflower ghee, rapeseed oil, fry light
21. Cakes & biscuits	11. Vegan cakes & biscuits	Cookies, Digestive twists, bourbons, Lotus Biscoff, vegan sponge cake, vegan cereal bars, party ring minis, granola bars
22. Sweets and desserts	12. Vegan sweets & desserts	Fudge, cheesecake pots, chocolate mousse pots, dark chocolate, non-dairy ice cream, churros, star burst sweets
23. Vegan crisps	13. Vegan crisps	Lentil Chips, Kettle chips, walkers, tortilla chips, vegetable chips, pretzel bites
24. Sauces & condiments	14. Sauces and condiments	BBQ sauce, cheese sauce, Red lasagne sauce, free from sauce, olive oil, vegetable oils, seeds, tahini, vegetable pates, mayonnaise, hummus, chocolate spread, coleslaw, potato salad
25. Salt	15. Salt	All added salts
26. Alcohol	16. Alcohol	Vegan friendly alcohols
27. Vegan takeaway	17. Vegan takeaway	From fast-food outlets providing vegan options
28. Cooking	18. Cooking from scratch	Additional question to help with establishing vegan patterns
29. Recipes used	19. Creating own recipes	Additional question to help with establishing vegan patterns
30. Use of vegan brands	20. Purchasing vegan brands	Additional question to help with establishing vegan patterns

### Factor analysis

Factor analysis with the principal component method was performed in SPSS, with the procedure ‘dimension reduction’ and ‘FACTOR’ on both sets of food groups to identify the primary components, which accounted for variation in dietary intake. However, the smaller set of food groups (*n* 20) was deemed more appropriate due to the small sample size^(35)^. The methods followed previous studies that have used factor analysis as a statistical method to reduce large sets of dietary intake variables into smaller sets of variables that represent eating patterns^(36,37)^. The smaller sets of composite variables derived through the principal component method are referred to as ‘components’, and the variables within these are referred to as ‘factors’. The Kaiser–Mayer–Olkin measure and Bartlett’s test of sphericity were undertaken before applying the principal component method, to ensure the data were suitable for factor analysis^(38)^. The twenty food variables from food groups 2 shown in Table 1 were entered into the factor analysis. Oblimin and Varimax rotations were applied. The components derived from the Oblimin rotation were selected similar to previous work by researchers exploring dietary patterns^(39,40)^. The rotation redistributes the variance of each component allowing for a simpler structure^(41)^. Oblimin rotation was chosen as the preferred method of ‘rotation’ as it has a range of advantages compared with other types of rotation^(42)^.

**Fig. 1:**
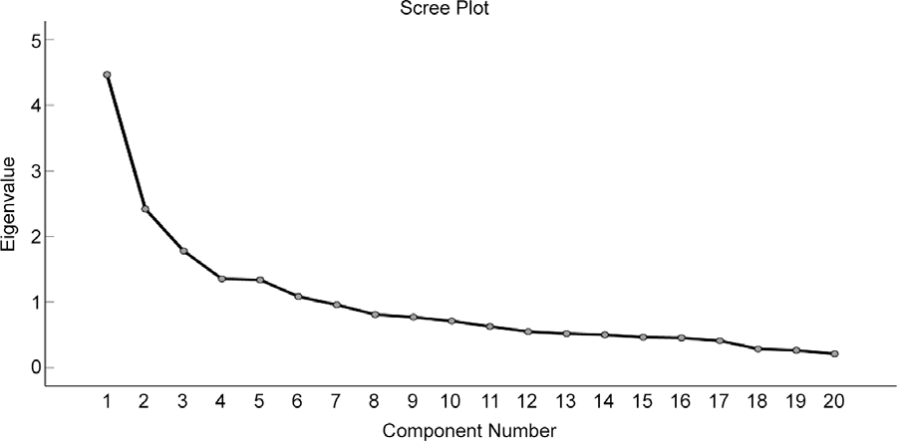
Scree plot to show eigenvalues of each component number

The number of components selected was based on the assessment of the scree plot, with values >1 deemed appropriate to establish the patterns that explain the largest proportion of variance^(36)^. Six components had an eigenvalue >1, but there was a gradual break in the scree plot after the fourth component (Fig. 1); therefore, four components were retained. The dietary patterns were characterised by high and low intakes of vegan food and drinks. The patterns were labelled based on the types of factors representing the component and explanations in the literature.

### Cluster analysis

Two-factor cluster analysis identifies groupings by running pre-clustering first and then by running hierarchical methods to enable automatic selection of the number of clusters^(35)^. Two-factor cluster analysis was performed to order the twenty food groups in a dendrogram, where food groups with the highest correlations were further grouped together, while samples with small correlations were widely separated. In particular, the two food groups with the largest correlation were identified and merged into a single ‘synthetic’ sample. The remaining food groups were then searched for the largest correlation with the synthetic sample. This process was repeated until all samples were merged into a single sample, and the correlations among samples were then expressed as a hierarchical tree^(43)^.

The dietary patterns were characterised by high and low intakes of vegan food and drinks. The clusters were labelled based on the types of inputs representing the component and explanations in the literature.

## Results

### Participant characteristics

Data collection took place from Monday 2 March 2020 through Friday 3 April 2020. There were 129 fully completed FFQ. Sample characteristics are presented in Table 2.

**Table 2:** Characteristics of study participants

	Sample (*n* 129)	Percentage
Age group (years)		
18–24	47	36
25–39	45	35
40–59	34	26
60–64	3	2
Sex		
Female	113	88
Male	16	13
Reason for adopting a vegan lifestyle		
Health	4	3
Environmental reasons	8	6
Health & environment	6	5
Animal welfare	28	22
Health & animal	7	5
Environment & animal	20	16
Health, environment & animal	56	43
Length of time following a vegan diet		
<6 months	22	17
6–12 months	10	8
1–3 years	53	41
4–10 years	30	23
Over 10 years	14	11
Taking nutritional supplements		
Vitamin B_12_	88	68
Vitamin D	54	42
*n*-3	18	14
Iodine	16	12
Fe	33	26
Ca	25	19
Zn	19	15
Se	10	8
Other	18	13

Most participants were female (87 %), and most were aged 18–24 (36 %) years. The most common reason selected for following a vegan lifestyle was ‘Health, Environment & Animal welfare’ (43 %). Health benefits were in the minority with only 3 % following the vegan lifestyle primarily for ‘health’. It is important to note that on the questionnaire, these were presented as separate reasons and not a single reason. Participants were able to select more than one reason. Most vegans (41 %) had been following a vegan diet for 1–3 years. Some participants (17 %) were eating a vegan diet for <6 months; (8 %) 6–12 months; (23 %) 4–10 years and (11 %) over 10 years. From those taking nutritional supplements, the majority took vitamin B_12_ (68 %). Almost half took vitamin D (42 %). A moderate number (26 %) were taking Fe supplements and 19 % took Ca supplements. A small number of participants (15 %, 12 %, 14 % and 7 %) consumed Zn, iodine, *n*-3 and Se supplements, respectively. Again these micronutrients were presented in a list on the questionnaire, and participants were able to select more than one supplement.

### Factor analysis

Inspection of the correlation matrix revealed the presence of many coefficients of 0·3 and above. The Kaiser–Meyer–Olkin value was 0·727, reaching the recommended value of 0·5^(38)^ The Barlett’s Test of Sphericity^(44)^ reached statistical significance, supporting the factorability of the correlation matrix^(35)^.

Factor analysis with the principal component method revealed the presence of six components with eigenvalues exceeding 1, explaining 22 %, 12 %, 9 %, 7 %, 7 % and 5 % of the variance. However, inspection of the scree plot (Fig. 1) revealed a gradual break after the fourth component. Therefore, the first four components explain the largest proportion of variance in the dietary intake data and were retained as ‘dietary patterns’. Together these components represent a cumulative percentage of 50 % of the inter-individual variability. To aid the interpretation of these four components, oblimin rotation was performed, representing four definite dietary patterns (Table 3). The first component in the matrix could be described as a ‘convenience pattern’ with high positive loadings for vegan sweets and desserts 0·802, vegan crisps 0·760, vegan sauces and condiments 0·591, vegan biscuits and cakes 0·536, fats and oils 0·49, vegan convenience meals & snacks 0·440 and dairy alternatives 0·363. For the second component, it was evident that the high positive loadings included cooking from scratch 0·846, creating recipes 0·785 and protein alternatives to meat/fish 0·445; this suggests a more health conscious vegan who is paying close attention to the types of foods in the vegan diet. The third component was characterised by high positive loadings for alcohol 0·800, takeaways 0·478 and salt 0·459. The fourth pattern was characterised by positive loadings for potatoes 0·849, vegetables 0·660, fruit 0·625 and refined grains 0·492. This pattern shares similarities to that of a traditional vegan definition. Much of the current literature supports that plant-based foods, fruit and vegetables are strongly associated with vegan eating^(24,45−47)^.

**Table 3:** Dietary patterns derived from factor analysis

Component matrix displaying factor loadings						
	Component					
	1	2	3	4	5	6
Vegan sauces & condiments	0·700					
Vegan sweets & desserts	0·671	−0·381				
Fruit	0·627			0·326		
Fats & oils	0·615			−0·385		
Vegan convenience meals & snacks	0·612	−0·376				
Vegan biscuits & cakes	0·557	−0·355				0·343
Refined grains	0·548			0·361		
Dairy alternatives	0·542			−0·402		
Protein alternatives to meat/fish	0·514	0·335				
Processed meat alternatives	0·497	−0·414			0·476	
Vegetables	0·467	0·461		0·320		
Cooking from scratch		0·827				
Creating your own recipe		0·752				
Salt			0·765			
Takeaways	0·336		0·558			
Alcohol			0·496			0·487
Potatoes	0·494		−0·384	0·506		
Purchasing vegan brands			0·343		0·553	−0·401
Vegan crisps	0·398				−0·507	
Whole grains	0·341		−0·302		0·339	0·518

Extraction method: principal component method.

### Cluster analysis

To further strengthen the findings from the factor analysis, two-factor cluster analysis was performed. The cluster analysis clearly identified the number of participants that represent each cluster and the percentage of participants who are regularly consuming the food items within each cluster. A cut-off point of 0·40 factor of importance was used to identify the most prevalent cluster groupings^(35)^. Two-factor cluster analysis indicated the presence of three different clusters. This analysis explained the groupings for 127 of the participants; two participants did not belong to any of the clusters. The clusters are categorised as shown in Table 4.

**Table 4:** Dietary patterns derived from cluster analysis

Cluster	3	1	2
Label	Health conscious	Unhealthy/convenience	Traditional/convenience
Description	Excluding most processed products. Fruit/veg/protein, alternatives to meat/fish & refined grains. Highest in cooking from scratch	Reliance on processed foods minimal preparation	Mainly consuming a traditional vegan diet but now trying some of the new vegan products on the market
Size of sample	*n* 65, 51·2 %	*n* 34, 26·8 %	*n* 28, 22·0 %
Input (predictor) importance	Inputs (mean)		
1·00	Vegan sauces and condiments 7·28	Vegan sauces and condiments 8·82	Vegan sauces and condiments 15·57
0·93	Vegan sweets and desserts 2·91	Vegan sweets and desserts 5·50	Vegan sweets and desserts 8·21
0·69	Vegan convenience meals & snacks 4·45	Vegan convenience meals & snacks 8·74	Vegan convenience meals & snacks 10·79
0·68	Fats and oils 14·03	Fats and oils 13·18	Fats and oils 21·29
0·59	Vegan biscuits and cakes 0·78	Vegan biscuits and cakes 2·44	Vegan biscuits and cakes 3·46
0·52	Vegan crisps 1·80	Vegan crisps 3·91	Vegan crisps 3·89
0·51	Creating your own recipe 2·78	Creating your own recipe 1·26	Creating your own recipe 3·68
0·47	Fruit 13·08	Fruit 18·00	Fruit 23·11
0·35	Vegetables 32·26	Vegetables 29·32	Vegetables 39·75
0·31	Dairy alternatives 3·28	Dairy alternatives 3·32	Dairy alternatives 5·86
0·29	Cooking from scratch 4·43	Cooking from scratch 3·29	Cooking from scratch 4·71
0·26	Refined grains 5·06	Refined grains 6·56	Refined grains 8·18
0·25	Potatoes 3·63	Potatoes 3·97	Potatoes 5·50
0·23	Potatoes 3·63	Potatoes 3·97	Potatoes 5·50
0·23	Alcohol 1·54	Alcohol 2·71	Alcohol 1·00
0·21	Protein alternatives to meat/fish 13·08	Protein alternatives to meat/fish 12·47	Protein alternatives to meat/fish17·07
0·17	Takeaways 0·80	Takeaways 1·09	Takeaways 1·50
0·15	Processed meat alternatives 4·46	Processed meat alternatives 6·09	Processed meat alternatives 6·50
0·08	Whole grains 3·25	Whole grains 3·53	Whole grains 4·82
0·06	Salt 2·88	Salt 3·21	Salt 3·89
0·03	Purchasing vegan brands 2·57	Purchasing vegan brands 2·91	Purchasing vegan brands 2·71

Cluster 1, ‘convenience’, representing 26·8 % (*n* 34) of the sample. Shows reliance on processed foods with minimum preparation for convenience perhaps because these are now readily available featuring vegan sauces & condiments, desserts, convenience meals/snacks & processed meat alternatives, refined grains. Also incorporating non-processed vegan foods (fruit, vegetables, fats and oils, protein alternatives to meat/fish). Foods that are quick and easy to prepare e.g. fruit/nuts. Possibly mindful of their protein intake having natural protein alternatives and ultraprocessed versions. This cluster lacks dairy alternatives.

Cluster 2, ‘traditional’, representing 22 % (*n* 28) of the sample. Mainly featuring traditional vegan foods, high amounts of fruit, vegetables, potatoes and wholegrains, however also with the most vegan convenience meals/snacks/sweets/desserts, fat and oils and dairy and protein alternatives. Perhaps representing those who are now trying some of the new vegan products on the market but are still health conscious enough to have their traditional balanced diet of protein, carbohydrates, fruit and vegetables.

Cluster 3, ‘health conscious’, representing 51·2 % (*n* 65) of the sample. The majority of the sample fit into this cluster. Vegans in this cluster are excluding most processed products, whilst opting for fruit/vegetable/protein alternatives to meat/fish and refined grains. There may be some potential for undereating; this cluster had the lowest mean values for dairy alternatives, fruit, whole and refined grains, vegan convenience meals/snacks/sweets/desserts and potatoes. This cluster could represent vegans following the diet for weight loss purposes or perhaps those who are committed to veganism for reasons outside of health/diet perhaps with less interest in food.

## Discussion

### Dietary patterns

**Fig. 2:**
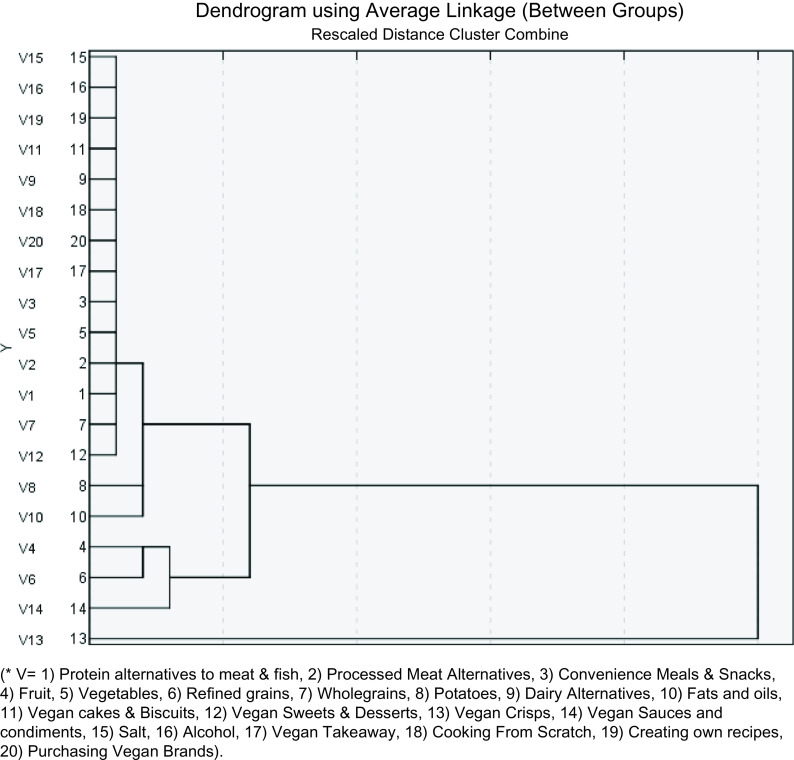
Dendrogram of dietary patterns derived from cluster analysis

Factor analysis with the principal component method identified four distinct dietary patterns outlined in Fig. 2 and Table 3, cumulatively accounting for 50 % of the total variance. The convenience dietary pattern was the most identifiable dietary behaviour to emerge from the analysis. It was characterised as a ‘Convenience’ pattern because the diet centred on vegan convenience meals and snacks, vegan sweets and desserts, sauces, condiments and fats. Similarly, the cluster analysis had two clusters focusing on processed vegan products such as convenience meals and snacks, sauces, condiments, desserts and processed meat alternatives. The association between these processed products is noteworthy considering the growth of veganism and the rapid rise in the production of vegan products^(3,48)^. A convenience pattern suggests that some respondents are using a range of processed vegan products, therefore not solely using natural ingredients to prepare meals. Similar findings were reported in South Asian vegetarians who use unhealthy convenience products^(49)^. The second component of factor analysis, represented vegans cooking from scratch and creating their own recipes whilst opting for natural protein sources such as soya and pulses over processed protein alternatives thus, component 2 could be described as the ‘Health Conscious’ dietary pattern. The current research demonstrates that vegans report cooking from scratch regularly irrespective of how long they have been vegan. Vegans of all age brackets report to ‘cook from scratch’ twice per week or more. It remains unknown what they are using to cook from scratch. This is important considering the most common dietary pattern was that of a convenience style pattern. To meaningfully address what vegans are cooking with, it will be necessary to refine the definition of ‘cooking from scratch’ in future questionnaires. Alternatively, the use of food diaries could further validate the findings of the FFQ. It is clear that the health-conscious group is cooking from scratch as well as eating more protein alternatives such as nuts, soya and legumes rather than ultra-processed alternatives such as meat-free burgers or bacon. The cluster analysis supported this recognising that some vegans (51 %) were consuming high intakes of fruit, vegetables and non-processed meat alternatives. Despite this healthy focus, there are still potential health issues as the cluster analysis also revealed these vegan diets had low intakes of dairy alternatives. It is unclear if this group was considering their micronutrient levels and taking nutritional supplements in place of dairy alternatives. In this study, not everyone was supplementing with vitamin B_12_ which is found mainly in animal products. To explain this, it is possible that individuals focused more on diet, to obtain specific nutrients from food, rather than using supplements. However, considering the vegan dietary patterns revealed in this study, another possible explanation could be that some vegans are not focusing on the nutritional quality of their diet. By way of illustration, less than half of the vegans in this study irrespective of motivation for veganism were supplementing their diet with key micronutrients such as iodine, Fe, Ca, Zn, Se and *n*-3. This suggests some vegan dietary patterns are not conducive to achieving recommended nutritional requirements. This is a particularly important question due to the vegan diet emerging as one of the most popular diet searches according to google trend^(4)^.

The third component of factor analysis constituted alcohol, vegan takeaways and salt. This component was comparable to an ‘unhealthy’ ‘takeaway’ dietary pattern that comprises processed meat alternatives that can still be high in salt. In this study, 36 % of respondents were aged 18–24 years limiting the generalisability of the patterns but perhaps this pattern could be related to student lifestyles. Although the respondents were not asked if they were students, previous studies have reported that students often have poor diets and binge drink alcohol, increasing their risk of disease^(50,51)^. The fourth component of factor analysis identified a ‘traditional’ vegan dietary pattern accounting for 7 % of total variance. Contrary to expectations, this pattern had the lowest variance in comparison with the other three. This pattern is in line with the typical vegan definition. The Vegan Society highlights that vegans follow strictly plant-based diets which exclude all animal products^(10)^. Yet, the small variance reflected from this pattern suggests that with the rise in vegan products, fewer vegans are following traditional vegan approaches, potentially compromising the nutritional quality of their diet. Espinosa-Marrón *et al.* ^(52)^ support this concept by acknowledging changes in eating habits, and food availability will affect the dietary choices that vegans make.

### The vegan food industry

A growing appetite for vegan foods has now gained the attention of the food industry. It is estimated the global value of meat alternative products will reach over £22 billion by 2023^(53)^. Our pattern analyses clearly indicate the vegan food industry is impacting vegan dietary choices. According to Fardet and Boirie^(54)^, the health benefits of plant-based diets are closely associated with the fact that such foods require the least amount of processing. In contrast, factor analysis in this study found that the main vegan dietary pattern was a convenience, ultraprocessed diet. Similarly, the third pattern identified represents unhealthy lifestyle behaviours featuring alcohol, takeaways and salt. Cluster analysis reveals clusters 1 and 2 (27 % and 22 % of the sample, respectively) comprised foods such as sauces, condiments, fats, processed meat alternatives and convenience foods. Together these findings are particularly concerning as they raise questions regarding the impact of ultraprocessed foods on the quality of some vegan diets.

Despite the growing number of people choosing to follow a vegan diet, there are still no specific official dietary guidelines for vegans in the UK. The Vegan Eatwell Guide is a relatively new resource that provides additional supportive information reinforcing key considerations for planning the diet. However, the unexpected findings from this study do not represent the Vegan Eatwell guide. Our cluster analysis showed that although cluster 3 (health conscious) represented most of the participants (51 %) and was made up of an array of healthy foods such as fruit, meat alternatives and vegetables, it did not consist of foods from each of the main food groups. For example, dairy alternative items did not factor at all in this cluster. The main dietary patterns presented in this study depict diets high in processed meat alternatives such as vegan burgers, nuggets, sausage rolls in contrast to natural plant-based proteins such as pulses, soya and tofu, which are recommended on the Vegan Eatwell guide.

### Potential concerns within vegan dietary patterns

Although full nutritional analysis was not conducted in this study, the findings from the factor and cluster analyses suggest some vegan diets are poorly constructed. Within this study, these findings warrant concern that some vegans may be at potential risk of nutritional deficiencies. Respondents were often on more than one supplement, although exact intakes were not recorded. The analysis revealed 68 % were supplementing one or more of the main nutrients of concern^(9)^ representing vitamin B_12_ (42 %), vitamin D (14 %), *n*-3 (12 %), iodine (26 %), Fe (19 %), Ca (15 %), Zn and Se (8 %).

Dairy alternatives were the only identified food group with potential to enhance B_12_ intakes; however, they only featured in the first identified dietary pattern for factor analysis and in the second cluster which represented only 22 % of the sample. Previous research has established that vegans consume sufficient amounts of dietary Fe, which prevents anaemia^(55)^. Food groups that could provide Fe in the vegan diet include vegetables, protein alternatives to meat/fish and refined (incorporating fortified white flour) grains^(55,56)^; however, these groups did not feature highly in any of identified clusters. The factor analysis also showed none of the identified patterns featured all of these food groups. Adequate consumption of fortified plant milks and soya products such as yoghurt can help vegans to meet dietary requirements for Ca; therefore, dietary adaptations are an important consideration to support bone health^(27)^. Dairy alternatives were a component in the ‘convenience’ factor analysis dietary pattern but not the other three and did not have a high predictor of importance for in the cluster analysis. This also has potential implications for iodine status in vegans. Cows’ milk is one of the best sources of iodine in the UK diet; however, with plant-based milks more popular than ever before, the UK population are at risk of mild iodine deficiency^(57,58)^. Vegans fitting the ‘convenience’ dietary pattern did incorporate dairy alternatives, thus potentially meeting iodine requirements. However, the alternative vegan dietary patterns warrant concern as they are all absent of dairy substitutes. This is particularly alarming as the majority of participants in this study were young females, who are thought to be particularly at risk of iodine deficiency in the UK^(59)^.

The dietary patterns and clusters ‘convenience’ and ‘unhealthy’ revealed in this study also warrant concern for *n*-3 status in vegans. It has been reported in the USA that some processed foods, meat substitutes and salad dressings have high quantities of *n*-6 linolenic acid present, which could further impair *n*-3 status^(60)^. Thus, nutritional data about the processed vegan products that have recently launched in the UK are urgently required.

In contrast to earlier findings, the dietary patterns found in this study suggest some vegan diets are highly processed with lower intakes of natural vegan foods. This is an important consideration especially as evidence reveals the level of processing can affect the nutritional quality of a food^(61,62)^. In light of the increasing numbers of people choosing to follow a vegan diet and the availability of ultraprocessed vegan food in the market, our findings suggest future studies examining vegan dietary patterns that incorporate nutritional and blood analysis into the study design should be a priority.

### Strengths and limitations

The evolution of a vegan diet when adapted to replace all animal foods with plant-based sources is important. This study is among the first to research the vegan diet specifically, identifying recent dietary patterns in a UK vegan cohort. It is unique for its distribution technique of social media, effectively recruiting a convenience sample to complete the FFQ. Vegan adaptation of the validated EPIC FFQ allowed participants to select from over 150 food items with a wide range of plant-based meat and dairy alternatives represented. Participants had the option to select ‘other’ ensuring a wide range of vegan food and drink items were captured. However, some limitations must be considered. The current analyses were based on a small convenience sample of 129 vegans, recruited through social media, which may affect the validity of the results. The recruitment phase was limited as the COVID-19 pandemic emerged in the UK. The research team was redeployed from their usual roles, and a decision was made to stop recruiting to the study to ensure sufficient time to analyse the data. To address demographical limitations, future studies should aim to increase the diversity of participants across gender and ethnicity, amend the inclusion criteria to vegans who have followed the diet for longer than 12 months and include more sociodemographical questions. Although the steps were taken to validate the adapted FFQ, further measures may help to enhance validity. Adapted FFQ are not compatible with Food Frequency Questionnaire European Prospective Investigation into Cancer and Nutrition Tool for Analysis (FETA) software; therefore, it may be more appropriate in future studies to ask a subsample of participants to complete a 3-d, 24 h weighed multiple pass recall (24 h MPR) outlining typical portion sizes to validate the FFQ responses^(63)^. This would allow future nutritional analysis similar to the work carried out in other studies^(23,39)^. It would have been interesting to perform blood analysis on the participants to compare the nutritional status within each of the identified dietary patterns. Future research utilising interviews could also explore why vegans eat what they do providing a more in depth insight into current vegan dietary patterns.

In conclusion, this study is the first to highlight the necessity of further investigations into vegan dietary patterns, particularly as there may be newly emerging dietary patterns that conflict with traditional vegan dietary patterns. If vegan dietary patterns are changing, it is prudent to consider the implications these new dietary choices may be having on health. Factor analysis identified four patterns within the vegan diet: (1) convenience; (2) health conscious; (3) unhealthy and (4) traditional in a cohort of 129 vegans. Whilst two healthy patterns were defined, the convenience pattern was the most identifiable pattern with a prominence of vegan convenience meals and snacks, vegan sweets and desserts, sauces, condiments and fats. Cluster analysis further strengthens these findings by confirming, that like the dietary patterns, the most predominant clusters consisted of an array of processed vegan food items. The association between these processed products is noteworthy considering the growth of veganism and the food industry’s response to this by providing a rapid rise in the production of vegan products.

Future research has potential to further verify our findings by collecting a proportion of weighed 24 h MPR from participants to determine exact portion sizes before undertaking nutritional analysis, following factor and cluster analyses. This research is a starting point but does raise some interesting questions regarding vegan dietary patterns, while the vegan food industry continues to grow. The findings from this small study have potential to shape and influence future vegan research. This novel study highlights the need for further vegan dietary pattern analysis studies that include nutritional and metabolic evaluation, particularly well-powered multicentre studies to ratify these results.

## Supplementary material

For supplementary material accompanying this paper visit https://doi.org/10.1017/S136898002100197X. click here to view supplementary material
