# Supplementary material for: Pattern analysis of vegan eating reveals healthy and unhealthy patterns within the vegan diet
Source: Public Health Nutr. 2021 May 11;25(5):1310–20. doi: 10.1017/S136898002100197X (PMC9991567; doi:10.1017/S136898002100197X)
Supplement: Supplementary file 1 [file S136898002100197Xsup001.doc]

Vegan Food Frequency Questionnaire

Page 1

*1.*  What made you decide to adopt a vegan lifestyle?

Health Benefits

Environmental Values

Religious Beliefs

Animal Welfare

To assist with weight loss

Other

*1.a.*  If you selected Other, please specify:

*2.*  What age category do you fit into:

18-24

25-39

40-59

60+

*3.*  How long have you been following a Vegan diet/ Lifestyle?

Less than 1 year

1-3years

4-10 years

Over 10 years

*4.*  What Gender are you?

Male

Female

Prefer not to say

*5.*  Do you take a nutritional supplement for any of the following

2 / 13

Vitamin B12

Vitamin D

Omega-3

Iodine

Iron

Calcium

Zinc

Selenium

Other

*5.a.*  If you selected Other, please specify:

*6.*  Do you feel your vegan diet is varied, providing you with a range of different foods across the week?

Yes

No

Not sure

*7.*  How often do you eat in vegan friendly cafes or restaurants?

Daily

Weekly

Monthly

Every 2- 6 months

Never

*8.*  Since following a Vegan diet do you feel you have spent more or less on food purchases?

Usually spend more

Usually spend less

Not noticed a difference

Not sure

*9.*  How best do you adapt your diet to ensure it is vegan?

Cook mostly from scratch using natural vegan ingredients

Rely mostly on vegan ready prepared meals and snacks

Use both natural vegan ingredients and vegan substitutes to create meals/snacks

Other

*9.a.*  If you selected Other, please specify:

*10.*  Do you eat baked vegan desserts/puddings/treats?

Yes I bake my vegan treats from scratch using vegan recipes

Yes I look for baked vegan treats that manufacturers provide

No I don’t eat these types of food

*11.*  How healthy do you think a vegan diet is for children under 16?

Very Healthy

Healthy

Quiet Healthy

Unhealthy

Not sure

*12.*  Please list your favourite 10 vegan brands (one per line)

 *Required*

1

2

3

4

5

6

7

8

9

10

*13.*  Please complete the following Food Frequency Questionnaire, and if possible please provide the names of the brands you commonly use. **Vegan Cereals**

|  | **Average Use Last Year** | | | | | | | | | Brand Name (optional) | | |
| --- | --- | --- | --- | --- | --- | --- | --- | --- | --- | --- | --- | --- |
|  | Never or less than once/month | 1-3 per month | Once a week | 2-4 per week | 5-6 per week | Once a day | 2-3 per day | 4-5 per day | 6+ per day |  |  |  |
| Weetabix Crispy Minis |  |  |  |  |  |  |  |  |  |  | | |
| Muesli cereal |  |  |  |  |  |  |  |  |  |  | | |
| Unicorn  Froot Loops |  |  |  |  |  |  |  |  |  |  | | |
| Fruit  Wheats |  |  |  |  |  |  |  |  |  |  | | |
| Crispy Rice |  |  |  |  |  |  |  |  |  |  |  |  |
| Shreddies |  |  |  |  |  |  |  |  |  |  |  |  |
| Frosted  Shreddies |  |  |  |  |  |  |  |  |  |  | | |
| Tesco Cinnamon Squares |  |  |  |  |  |  |  |  |  |  | | |
| Tesco free from crispy choco snaps |  |  |  |  |  |  |  |  |  |  | | |
| Aldi/Harvest Moon Multi- Grain  Hoops |  |  |  |  |  |  |  |  |  |  | | |

*14.*  **Fruit and Vegetables**: 1 medium size/ Handful/80g portion

|  | **Average Use Last Year** | | | | | | | | |
| --- | --- | --- | --- | --- | --- | --- | --- | --- | --- |
|  | Never or less than once/month | 1-3 per month | Once a week | 2-4 per week | 5-6 per week | Once a day | 2-3 per day | 4-5 per day | 6+ per day |
| Apple |  |  |  |  |  |  |  |  |  |
| Banana |  |  |  |  |  |  |  |  |  |
| Grapes |  |  |  |  |  |  |  |  |  |
| Plums |  |  |  |  |  |  |  |  |  |
| Kiwi |  |  |  |  |  |  |  |  |  |
| Orange |  |  |  |  |  |  |  |  |  |
| Peach |  |  |  |  |  |  |  |  |  |
| Pineapple |  |  |  |  |  |  |  |  |  |
| Mango |  |  |  |  |  |  |  |  |  |

| Papaya |  |  |  |  |  |  |  |  |  |
| --- | --- | --- | --- | --- | --- | --- | --- | --- | --- |
| Raspberries |  |  |  |  |  |  |  |  |  |
| Blueberries |  |  |  |  |  |  |  |  |  |
| Strawberries |  |  |  |  |  |  |  |  |  |
| Blackberries |  |  |  |  |  |  |  |  |  |
| Dried Fruit (Raisins or Dates or prunes etc.) |  |  |  |  |  |  |  |  |  |
| Avocado |  |  |  |  |  |  |  |  |  |
| Fruit Juice |  |  |  |  |  |  |  |  |  |
| Fruit Smoothie |  |  |  |  |  |  |  |  |  |
| Carrots |  |  |  |  |  |  |  |  |  |
| Cabbage |  |  |  |  |  |  |  |  |  |
| Spinach |  |  |  |  |  |  |  |  |  |
| Broccoli |  |  |  |  |  |  |  |  |  |
| Onion |  |  |  |  |  |  |  |  |  |
| Mushroom |  |  |  |  |  |  |  |  |  |
| Peppers |  |  |  |  |  |  |  |  |  |
| Tomatoes |  |  |  |  |  |  |  |  |  |
| Salad Leaves |  |  |  |  |  |  |  |  |  |
| Pulses (peas/beans/lentils) |  |  |  |  |  |  |  |  |  |
| Sweetcorn |  |  |  |  |  |  |  |  |  |
| Beetroot |  |  |  |  |  |  |  |  |  |
| Leeks |  |  |  |  |  |  |  |  |  |
| Cucumber |  |  |  |  |  |  |  |  |  |

*15.*  **Potatoes, Rice, Pasta**

**Average Use Last Year**

Never or less than once/month

1-3 per month

Once a week

2-4 per week

5-6 per week

Once a day

2-3 per day

4-5 per day

6+ per day

Brand Name (optional)

White Rice

Brown Rice

Wild Rice

Rainbow Rice

Broccoli Rice

Cauliflower Rice

Quinoa

White Bread

Seeded bread

Wholemeal bread

Beetroot/Spinach

Wraps

Asda falafel & spiced houmos sandwich

M&S super greens sandwich

M&S Rainbow vegetable sandwich

On the go sainsburys vegan sandwich range

URBANeat’s prepacked vegan sandwich

Pret a Manger’s super green and reds sandwich

Subway veggie Delite (vegan bread option)

Other vegan sandwich (please state in brand section)

Asda onion bhaji wrap

Café Nero houmous & falafel wrap

Tesco falafel & Houmous wrap

URBANeat’s Bombay spiced veggie wrap

Other vegan wraps (please state in brand section)

Vegan Pizza- Plantkitchen

Goodfellas vegan pizza

Pizza Express vegan giardiniera

The white rabbit

–vegan pizza

Sainsbury’s love your veg- vegan pizza

Other (please state in brand section)

Vegan Garlic bread slices (please state in brand section)

Couscous

Spaghetti

Lasagne sheets

Packaged pasta

Potatoes

Sweet potatoes

Ready prepared potatoes (Mash)

Vegan French

Fries

Plant kitchen

Dirty Fries

Pre packed

Roast Potatoes

*16.*  **Vegan Meat & Fish Alternatives**

|  | Average Use Last Year | | | | | | | | | Brand Name (Optional) | | |
| --- | --- | --- | --- | --- | --- | --- | --- | --- | --- | --- | --- | --- |
|  | Never or less than once/month | 1-3 per month | Once a week | 2-4 per week | 5-6 per week | Once a day | 2-3 per day | 4-5 per day | 6+ per day |  |  |  |
| Meet free mince |  |  |  |  |  |  |  |  |  |  | | |
| Meat free sausages |  |  |  |  |  |  |  |  |  |  | | |
| Bacon |  |  |  |  |  |  |  |  |  |  |  |  |
| Burgers |  |  |  |  |  |  |  |  |  |  |  |  |
| Chorizo |  |  |  |  |  |  |  |  |  |  |  |  |

| Ham  Slices |  |  |  |  |  |  |  |  |  |  | | |
| --- | --- | --- | --- | --- | --- | --- | --- | --- | --- | --- | --- | --- |
| Turkey  Slices |  |  |  |  |  |  |  |  |  |  | | |
| Chicken  Nuggets |  |  |  |  |  |  |  |  |  |  | | |
| Sausage  Rolls |  |  |  |  |  |  |  |  |  |  | | |
| Vegan  Pasty |  |  |  |  |  |  |  |  |  |  | | |
| No Fish  Fingers |  |  |  |  |  |  |  |  |  |  | | |
| Soya |  |  |  |  |  |  |  |  |  |  |  |  |
| Tempeh |  |  |  |  |  |  |  |  |  |  |  |  |
| Tofu  Pieces |  |  |  |  |  |  |  |  |  |  | | |
| Silken tofu |  |  |  |  |  |  |  |  |  |  | | |
| Lentils |  |  |  |  |  |  |  |  |  |  |  |  |
| Beans |  |  |  |  |  |  |  |  |  |  |  |  |
| Peas |  |  |  |  |  |  |  |  |  |  |  |  |
| Falafel |  |  |  |  |  |  |  |  |  |  |  |  |
| Vegan Ready Meals |  |  |  |  |  |  |  |  |  |  | | |
| Other products that you consume: |  |  |  |  |  |  |  |  |  |  | | |

*17.*  **Vegan Soups, Sauces & Spreads**

|  | Average use last year | | | | | | | | | Brand Name (optional) |
| --- | --- | --- | --- | --- | --- | --- | --- | --- | --- | --- |
|  | Never or less than once/month | 1-3 per month | Once a week | 2-4 per week | 5-6 per week | Once a day | 2-3 per day | 4-5 per day | 6+ per day |  |
| Free + easy Vegan Soups |  |  |  |  |  |  |  |  |  |  |
| Inspired Vegan Tartare sauce |  |  |  |  |  |  |  |  |  |  |
| BBQ  sauce |  |  |  |  |  |  |  |  |  |  |

| cheese sauce |  |  |  |  |  |  |  |  |  |  | | |
| --- | --- | --- | --- | --- | --- | --- | --- | --- | --- | --- | --- | --- |
| Red lasagne sauce |  |  |  |  |  |  |  |  |  |  | | |
| Free from white lasagne sauce |  |  |  |  |  |  |  |  |  |  | | |
| Free from tomato and basil pasta bake sauce |  |  |  |  |  |  |  |  |  |  | | |
| Salad  Dressings |  |  |  |  |  |  |  |  |  |  | | |
| Tahini |  |  |  |  |  |  |  |  |  |  |  |  |
| Vegetable pates |  |  |  |  |  |  |  |  |  |  | | |
| Houmous |  |  |  |  |  |  |  |  |  |  |  |  |
| Mayo |  |  |  |  |  |  |  |  |  |  |  |  |
| Peanut butter |  |  |  |  |  |  |  |  |  |  | | |
| Marmite |  |  |  |  |  |  |  |  |  |  |  |  |
| Coconut spread |  |  |  |  |  |  |  |  |  |  | | |
| Chocolate  Spread |  |  |  |  |  |  |  |  |  |  | | |
| Coleslaw |  |  |  |  |  |  |  |  |  |  |  |  |
| Potato  Salad |  |  |  |  |  |  |  |  |  |  | | |
| Other |  |  |  |  |  |  |  |  |  |  |  |  |

*18.*  **Dairy & Fat Alternatives**

|  | Average Use Last Year | | | | | | | | | Brand Name (optional) | | |
| --- | --- | --- | --- | --- | --- | --- | --- | --- | --- | --- | --- | --- |
|  | Never or less than once/month | 1-3 per month | Once a week | 2-4 per week | 5-6 per week | Once a day | 2-3 per day | 4-5 per day | 6+ per day |  |  |  |
| Soy Milk |  |  |  |  |  |  |  |  |  |  |  |  |
| Almond  Milk |  |  |  |  |  |  |  |  |  |  | | |
| Oat Milk |  |  |  |  |  |  |  |  |  |  |  |  |

| Rice Milk |  |  |  |  |  |  |  |  |  |  |  |  |
| --- | --- | --- | --- | --- | --- | --- | --- | --- | --- | --- | --- | --- |
| Coconut  Milk |  |  |  |  |  |  |  |  |  |  | | |
| Other Plant based milks: |  |  |  |  |  |  |  |  |  |  | | |
| Nutritional  Yeast |  |  |  |  |  |  |  |  |  |  | | |
| Vegan hard cheese |  |  |  |  |  |  |  |  |  |  | | |
| Cottage  Cheese |  |  |  |  |  |  |  |  |  |  | | |
| Not- zzarella sticks |  |  |  |  |  |  |  |  |  |  | | |
| Yoghurt alternatives |  |  |  |  |  |  |  |  |  |  | | |
| Custard alternatives |  |  |  |  |  |  |  |  |  |  | | |
| Olive Oil |  |  |  |  |  |  |  |  |  |  |  |  |
| Sunflower  Oil |  |  |  |  |  |  |  |  |  |  | | |
| Coconut  Oil |  |  |  |  |  |  |  |  |  |  | | |
| Canola Oil |  |  |  |  |  |  |  |  |  |  |  |  |
| Avocado  Oil |  |  |  |  |  |  |  |  |  |  | | |
| Sunflower  Ghee |  |  |  |  |  |  |  |  |  |  | | |
| Olive Ghee |  |  |  |  |  |  |  |  |  |  |  |  |
| Vegan butter alternative |  |  |  |  |  |  |  |  |  |  | | |
| Nutivia- organic coco oil |  |  |  |  |  |  |  |  |  |  | | |
| Other  Products: |  |  |  |  |  |  |  |  |  |  | | |

*19.*  **Vegan Sweets & Snacks**

|  | Average Use Last Year | | | | | | | | | Brand Name (optional) |
| --- | --- | --- | --- | --- | --- | --- | --- | --- | --- | --- |
|  | Never or less than once/month | 1-3 per month | Once a week | 2-4 per week | 5-6 per week | Once a day | 2-3 per day | 4-5 per day | 6+ per day |  |

| Nuts |  |  |  |  |  |  |  |  |  |  |  |  |
| --- | --- | --- | --- | --- | --- | --- | --- | --- | --- | --- | --- | --- |
| Seeds |  |  |  |  |  |  |  |  |  |  |  |  |
| Fudge |  |  |  |  |  |  |  |  |  |  |  |  |
| Lotus Biscoff biscuits |  |  |  |  |  |  |  |  |  |  | | |
| Carrot cake  Oreo |  |  |  |  |  |  |  |  |  |  | | |
| Digestive  Twists |  |  |  |  |  |  |  |  |  |  | | |
| Cookies |  |  |  |  |  |  |  |  |  |  |  |  |
| Vegan Cheesecake pots |  |  |  |  |  |  |  |  |  |  | | |
| Chocolate  Mousse pot |  |  |  |  |  |  |  |  |  |  | | |
| Doughnuts |  |  |  |  |  |  |  |  |  |  |  |  |
| Sponge cake |  |  |  |  |  |  |  |  |  |  | | |
| Classic dark chocolate |  |  |  |  |  |  |  |  |  |  | | |
| Made  without dairy chocolate |  |  |  |  |  |  |  |  |  |  | | |
| Non Dairy  Ice cream |  |  |  |  |  |  |  |  |  |  | | |
| Vego bars |  |  |  |  |  |  |  |  |  |  |  |  |
| Nakd bars |  |  |  |  |  |  |  |  |  |  |  |  |
| Gronola  Bars |  |  |  |  |  |  |  |  |  |  | | |
| Skittles |  |  |  |  |  |  |  |  |  |  |  |  |
| Party ring minis |  |  |  |  |  |  |  |  |  |  | | |
| Starburst sweets |  |  |  |  |  |  |  |  |  |  | | |
| Vegetable  Chips |  |  |  |  |  |  |  |  |  |  | | |
| Vegan crisps |  |  |  |  |  |  |  |  |  |  | | |
| Lentil Chip |  |  |  |  |  |  |  |  |  |  |  |  |
| Churros |  |  |  |  |  |  |  |  |  |  |  |  |
| Pretzel bites |  |  |  |  |  |  |  |  |  |  |  |  |

*20.*  **Vegan Eating Patterns**

|  | Average Use Last Year | | | | | | | | |
| --- | --- | --- | --- | --- | --- | --- | --- | --- | --- |
|  | Never or less than once/month | 1-3 per month | Once a week | 2-4 per week | 5-6 per week | Once a day | 2-3 per day | 4-5 per day | 6+ per day |
| How often do you buy Vegan branded food or drink products? |  |  |  |  |  |  |  |  |  |
| How often do you cook from scratch? |  |  |  |  |  |  |  |  |  |
| How often do you create your own vegan recipes? |  |  |  |  |  |  |  |  |  |
| How often to you purchase Vegan take away? |  |  |  |  |  |  |  |  |  |
| How often do you drink alcohol? |  |  |  |  |  |  |  |  |  |

Page 2: Thank you

Thank you for completing this survey
